# Challenges experienced with early introduction and sustained consumption of allergenic foods in the Enquiring About Tolerance (EAT) study: A qualitative analysis

**DOI:** 10.1016/j.jaci.2019.09.004

**Published:** 2019-12

**Authors:** Paula Voorheis, Sadie Bell, Laura Cornelsen, Matthew Quaife, Kirsty Logan, Tom Marrs, Suzana Radulovic, Joanna Craven, Carsten Flohr, Gideon Lack, Michael R. Perkin, Enquiring About Tolerance (EAT) study team, Louise Young, Victoria Offord, Mary DeSousa, Jason Cullen, Katherine Taylor, Anna Tseng, Bunmi Raji, Sarah Byrom, Gillian Regis, Charlie Bigwood, Charlotte Stedman, Sharon Tonner, Emily Banks, Yasmin Kahnum, Rachel Babic, Ben Stockwell, Erin Thompson, Lorna Wheatley, Devi Patkunam, Kerry Richards, Ewa Pietraszewicz, Alick Stephens, Asha Sudra, Victor Turcanu

**Affiliations:** a Executive Office, London School of Hygiene and Tropical Medicine, London, United Kingdom; b Faculty of Public Health and Policy, London School of Hygiene and Tropical Medicine, London, United Kingdom; c Paediatric Allergy Research Group, Department of Women and Children's Health, School of Life Course Sciences, King's College London, London, United Kingdom; d Unit for Population-Based Dermatology Research, St John's Institute of Dermatology, School of Basic and Medical Biosciences, Faculty of Life Sciences & Medicine, King's College London, London, United Kingdom; e Population Health Research Institute, St George's, University of London, London, United Kingdom

**Keywords:** Food allergy, diet, allergens, infancy, breastfeeding, randomized controlled trial, adherence, qualitative, EAT, Enquiring About Tolerance, EIG, Early introduction group, RR, Risk ratio, UK, United Kingdom

## Abstract

**Background:**

The early introduction group participants of the Enquiring About Tolerance study were asked to undertake a proscriptive regimen of early introduction and sustained consumption of 6 allergenic foods. It was envisaged that this might be challenging, and early introduction group families were presented with an open-text question to express any problems they were experiencing with the regimen in recurring online questionnaires.

**Objective:**

We sought to analyze these open-text questionnaire responses with the aim of identifying challenges associated with the introduction and regular consumption of allergenic foods.

**Methods:**

Three combinations of interim questionnaire responses were selected for analysis, representing the early period (4, 5, and 6 months), middle period (8 and 12 months), and late period (24 and 36 months) of participation in the Enquiring About Tolerance study. Responses were assigned a code to describe their content and subsequently grouped into themes to portray key messages. A thematic content analysis allowed for conversion of qualitative codes into quantitative summaries.

**Results:**

Three main challenges to allergenic food consumption were identified. First, some children refused the allergenic food, causing a sense of defeat among caregivers. Second, caregivers were concerned that allergenic foods might be causing a reaction, triggering a need for reassurance. Third, practical problems associated with the regimen compromised caregivers' capacity to persist.

**Conclusion:**

Understanding the challenges experienced with allergenic food introduction and sustained consumption is the necessary precursor to developing specific communication and support strategies that could be used by caregivers, practitioners, policymakers, and key stakeholders to address these problems.

## Graphical abstract

**Figure:**
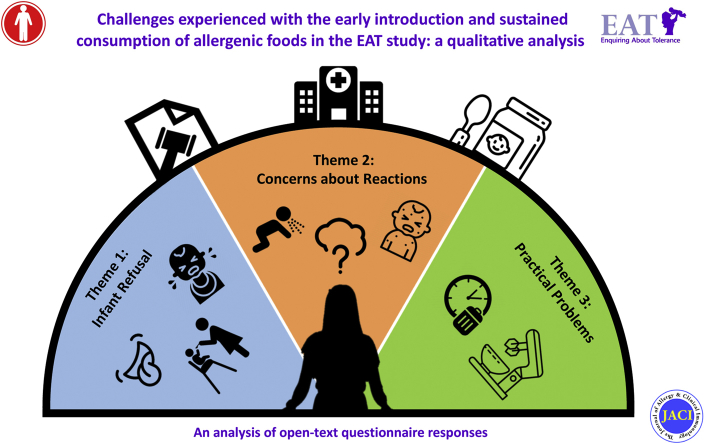

Childhood food allergies are a growing public health concern, with their prevalence reaching as high as 10% in developed countries.^1^ Although clinical practice guidelines historically advised against introducing allergenic foods in early infancy, recent evidence supports early introduction of allergenic foods to prevent food allergies.^2^, ^3^, ^4^, ^5^, ^6^, ^7^ Although several countries have amended their infant feeding guidelines to reflect this new evidence,^8^, ^9^, ^10^ concerns remain about the acceptability and efficacy of new guidance in practice. A recent survey of new and expectant caregivers of infants at risk for peanut allergy found poor willingness and questionable support for early allergenic solid food introduction.^11^

In the Enquiring About Tolerance (EAT) study (International Standard Randomized Controlled Trial no. register: ISRCTN14254740),^2^ exclusively breastfed infants from the general population were randomized to introduce 6 allergenic foods from 3 months of age in the early introduction group (EIG) or to exclusively breastfeed to around 6 months, with allergenic food introduction beyond this point being at parental discretion in the standard introduction group. Adherence in the EIG was low, with only 42.2% (223/529) of adherence-evaluable EIG participants being per-protocol adherent.

After completion of the EAT study, we have undertaken a quantitative analysis of the enrollment and postenrollment factors influencing adherence within the key early introduction period (up to 6 months of age) in EIG families.^12^ Poor adherence was associated with older maternal age, nonwhite ethnicity, and lower maternal quality of life at enrollment.^12^ After enrollment, parent-reported IgE-type symptoms and reported feeding difficulties by 4 months of age were also significantly associated with nonadherence.^12^

Although these findings were helpful in determining who was less likely to adhere, the reasons why early introduction and sustained consumption of allergenic foods proved to be challenging remained unknown. In each interim questionnaire sent every month between 4 and 12 months of age and every 3 months between 12 and 36 months of age, EIG families completed an open-text question about problems they were experiencing with their infant consuming the early introduction foods. In this article we have qualitatively analyzed these open-text responses with the aim of gaining a deeper understanding of the challenges involved with the introduction and ongoing consumption of allergenic foods.

## Methods

In the EAT study 1303 three-month-old infants were recruited from the general population in England and Wales. Children were enrolled between November 2009 and July 2012 and were healthy, exclusively breastfed, and born at term. After randomization, participants in the EIG who had negative results on skin prick tests or positive results on skin prick tests but negative results on food challenges were asked to start feeding their infants the 6 allergenic foods.

A multifaceted approach was used to support EIG families with undertaking the early introduction regimen that included providing dietician advice booklets, dietician-produced videos, automatic alert e-mails to EAT dieticians if participants reported problems, and the EAT study team's contact details (described in more detail in the Methods section in this article's Online Repository at www.jacionline.org).

The definitions of overall per-protocol adherence are provided in Table E1 in this article's Online Repository at www.jacionline.org. The key criterion for overall adherence in the EIG was consumption of at least 5 of the allergenic foods in at least 75% of the recommended amount (3 g of allergen protein/wk) for at least 5 weeks between 3 and 6 months of age.

The primary outcome was challenge-proven food allergy to 1 or more of the 6 early introduction foods between 1 and 3 years of age. Ethical approval for the EAT study was provided by St Thomas' Hospital Research Ethics Committee (Research Ethics Committee reference no. 08/H0802), and informed consent was obtained from the parents of all children enrolled in the study.

### Data collection

Each interim questionnaire was sent to all 652 EIG participants. The overall questionnaire response rate diminished over time, with the exception of the 12- and 36-month interim questionnaires, which had much higher response rates caused by coinciding clinical visits (see Fig E1 in this article's Online Repository at www.jacionline.org). On each interim questionnaire, EIG participants were asked to record the degree to which they were adhering to the consumption target of 4 g of each allergenic food protein per week supported by a guideline table. Adherence was instantaneously evaluated, and if consumption for 1 or more foods was 50% or less than the recommended amount, an alert was produced (described in more detail in the Methods section in this article's Online Repository). Regardless of whether the alert had been generated, the next question was an open-text question that asked for brief details about any particular problems caregivers were having with introducing the allergenic foods in that month: “If you have had a particular problem with your baby consuming the foods over the past month please provide brief details in the following box.” The question was not mandatory to fill in, and EIG respondents could leave it blank. Hence for any individual interim questionnaire, the EIG participants could be divided into 3 groups: EIG families who had not completed the questionnaire at all (designated “nonresponders to questionnaire”), EIG families who had completed the questionnaire but left the problem question blank (designated “problem question left blank”), and EIG families who had completed the questionnaire and entered a text response in the problem question box (designated “text response entered”).

If a textual response had been entered as “not applicable” or “n/a,” these participants were combined with the “problem question left blank” group because it could not be inferred with certainty whether this meant the family was experiencing no problems with allergenic food introduction. If participants entered text that actively described them having no problems with introduction, this was allocated into a “no problems” category.

### Data analysis

Three combinations of interim questionnaire responses to this question were selected for analysis: early period (4, 5, and 6 months; the key period for defining per-protocol adherence), middle period (8 and 12 months), and late period (24 and 36 months). For each time period analyzed, open-text responses were assigned a code to describe their content. Codes were then sorted into categories by moving related codes together. Finally, categories were grouped into themes to allow for the expression of key messages from the data. Using a thematic content analysis allowed for conversion of inductively derived codes into quantitative summaries through counting the responses in each thematic grouping. Reviewing the responses over time allowed overarching patterns to be determined.

The 3 factors found to be associated with nonadherence in our quantitative analysis article were as follows: older maternal age (greater than or equal to the median maternal age of 33 years), nonwhite ethnicity (vs white ethnicity), and lower maternal quality of life at enrollment (less than the median WHO Quality of Life–BREF score in the psychological domain at enrollment). We assessed whether these characteristics altered the likelihood of reporting identified themes. Risk ratios (RRs) were calculated for the early period among EIG families that had completed all 3 interim questionnaires (4, 5, and 6 months), and a count was made as to whether the family had mentioned a specific theme in 1 or more of the 3 questionnaires. Data were stored and analyzed in Microsoft Excel (Microsoft, Redmond, Wash).

## Results

### Participants

The characteristics of the participants can be seen in Table E2 in this article's Online Repository at www.jacionline.org. Of the 652 EIG participants, 85% were of a white ethnicity, 58% were greater than or equal to the mean age of 33 years, and 34% achieved adherence.

### Response rate and per-protocol adherence

**Fig 1:**
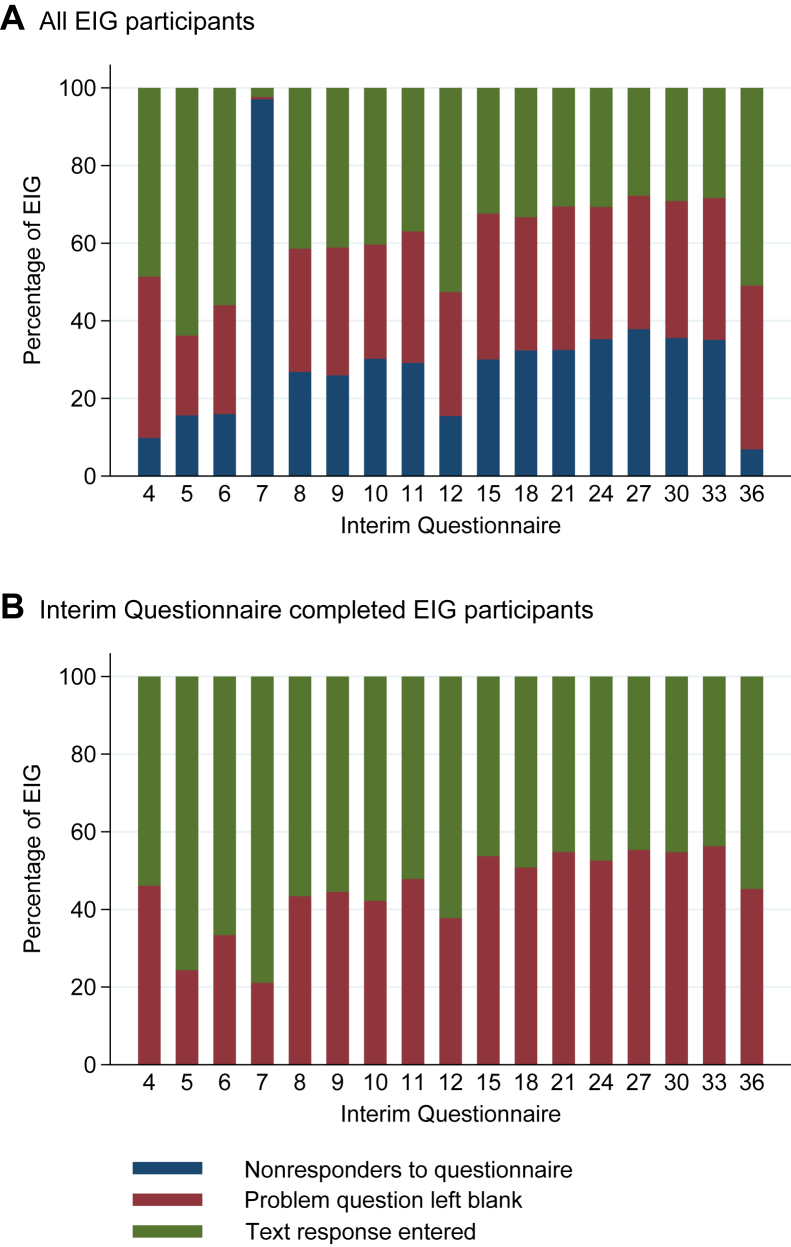
Problem question response types across all interim questionnaires. Relative distribution of the 3 possible response types at each interim questionnaire time point is demonstrated. For all EIG participants **(A)**, the proportion giving a text response diminished over time *(green bar)*. Over time, the proportion of EIG families not completing the questionnaire increased significantly *(blue bar)*, except for the 12- and 36-month interim questionnaires, which were completed in conjunction with a clinical visit. There was a routing issue within the 7-month interim questionnaire, which meant that the majority of EIG families who completed the questionnaire were inadvertently not offered the problem question. If EIG families who did not complete the interim questionnaire are excluded, it can be seen **(B)** that there is a steady increase over time in EIG families leaving the problem question blank *(red bar)* with a concomitant decrease in those entering a response *(green bar)*.

Over time, EIG families were more likely to not complete the interim questionnaire (Fig 1, *A*). Among those EIG families who completed the interim questionnaire, it can be seen that the proportion entering a text response to the problem question diminished over time, with a concomitant increase in those leaving it blank (Fig 1, *B*). Comparing adherent and nonadherent EIG participants, in the initial months significantly more nonadherent families were recording a response to the problem question, but the difference between the adherent and nonadherent groups diminished over time (data not shown).

Although some families repeatedly provided responses to the problem question and others persistently left it blank, there was considerable flux between the 3 groups of responses (text response entered, problem question left blank, or no data because questionnaire not completed; see Fig E2 in this article's Online Repository at www.jacionline.org).

Participants who left the problem question blank demonstrated per-protocol adherence rates that were significantly greater than those of participants who entered a text response, whereas participants who did not respond to the questionnaire at all had per-protocol adherence rates that were consistently lower than those of either group, although there was a relatively small number of questionnaire nonresponders in whom per-protocol adherence could be evaluated (Table I). This suggests that EIG participants who left the problem question blank might have been doing so because they were having significantly fewer problems with the early introduction regimen and did not find it necessary to provide a response.

**Table I:** Per-protocol adherence rates by month within the early, medium, and late periods for the 3 groups of those who did not complete the interim questionnaire, those who left the problem question blank, and those entering a response to the problem question

Period	Month	Nonresponse to questionnaire		Problem question left blank		Response entered		Total
		No.	Per-protocol adherence, % (n/N)	No.	Per-protocol adherence, % (n/N)	No.	Per-protocol adherence, % (n/N)	
Early period	4	64	30.8 (4/13)∗	271	45.0 (104/231)	317	40.4 (115/285)	652
	5	102	0 (0/5)∗	134	57.8 (74/128)	416	37.6 (149/396)	652
	6	104	0 (0/9)∗	183	61.8 (105/170)	365	33.7 (118/350)	652
Middle period	8	175	24.1 (19/79)	207	50.8 (97/191)	270	41.3 (107/259)	652
	12	101	39.5 (17/43)	208	48.9 (88/180)	343	38.6 (118/306)	652
Late period	24	230	35.9 (47/131)	222	43.0 (89/207)	200	45.6 (87/191)	652
	36	45	33.3 (6/18)	275	43.2 (98/227)	332	41.9 (119/284)	652

∗ Determining per-protocol adherence was dependent on responses to the key 4-, 5-, and 6-month questionnaires. Hence it was only possible to determine the per-protocol adherence rate in a small proportion of those EIG families who had not completed 1 or more of the 4-, 5-, and 6-month questionnaires. In the early period, among the small number of nonresponders in whom adherence could be determined, per-protocol adherence rates were consistently lower in this group compared with those who left the problem question blank and those who entered a text response.

### Word cloud

**Fig 2:**
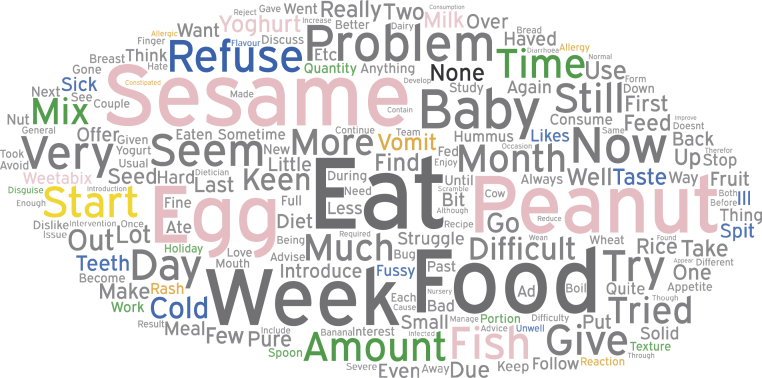
A word cloud of the most frequent words used in the open-text responses in all interim questionnaires analyzed. Depicted in this word cloud are the top 200 words that were most frequently used, excluding common words and combining like words (eg, sesame and tahini combined as sesame). The size of the words corresponds to the frequency with which they were used in the responses analyzed. Words that depict the main themes identified within the qualitative analysis of the open-text responses are color coded according to theme (blue, infant refusal; orange, reaction concerns; green, practical problems; yellow, issues with starting; and black, no problems). The 6 early introduction foods are identified in pink, and their relative size gives a clear indication of the frequency with which the specific food featured in the open-text responses.

A word cloud, which displays the most frequent words written within the text, can be seen in Fig 2. In addition to displaying raw text data, the word cloud demonstrates the frequency with which specific introductory foods feature in the responses and suggests that the propensity to make comments was food specific. Although families were responding to a question asking about specific problems they were having with introducing the allergenic foods, they often included comments about foods that were being consumed successfully. Hence the size of specific words in the word cloud represents a combination of predominantly problem-related entries but some positive entries as well.

### Themes

**Fig 3:**
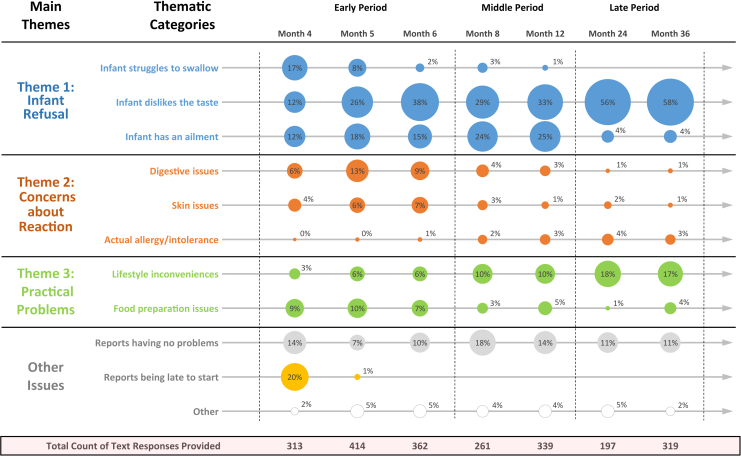
A bubble chart displaying the proportion of text responses coded into a thematic category within each interim questionnaire grouped by period. In each interim questionnaire *(columns)*, all text responses were coded to represent a thematic category *(rows)*. Sizes of bubbles are proportional to percentages of responses in any individual interim questionnaire that were coded to a particular theme. The variation in bubble size within and across months shows how the dominance of thematic categories can change in relation to the other thematic categories and in relation to themselves over time.

Three fundamental themes that summarize the challenges experienced during allergenic food introduction and ongoing consumption were identified in the open-text responses: infant food refusal, concerns about reactions and intolerances, and practical problems. Fig 3 is a longitudinal bubble chart that displays the proportion of text responses representing each thematic category within the interim questionnaires. The variation in bubble size shows how the dominance of themes changes across months and time periods.

#### Theme 1: Infant refusal

Caregivers often reported their infant rejecting the introduction foods. The main reasons for infant refusal have been categorized as (1) the infant struggles to swallow the food, (2) the infant dislikes the taste of the food, and (3) the infant has an ailment preventing consumption. Caregivers tended to feel increasingly defeated and at a loss as to how to manage this. This was the predominant theme expressed in the open-text responses across all 3 time periods (Fig 3).

At the beginning of the study (Fig 3; month 4, theme 1), the infants' gagging, spitting, or pushing the food back out made it physically challenging to introduce adequate amounts of allergenic food. This aversive behavior was immediately followed by emergent taste preferences (Fig 3; months 5-6, theme 1), which were often at odds with the allergenic food. Although this was challenging, caregivers initially remained positive about persevering: “Initially we had problems with the tastes of certain foods i.e. egg, so are having to find ways to make them taste nicer for her!” (month 5, nonadherent caregiver).

However, because taste preferences continued to dominate throughout the study (Fig 3; middle period and late period, theme 1), a growing sense of defeat among caregivers became evident. Childhood fussy eating behavior also became a problem, in which general inconsistences around eating habits became a barrier to maintaining consistency in allergenic food consumption: “Becoming fussy with food. Struggling to get food into her, both intervention foods and others. No 2 days are the same at present” (month 24, adherent caregiver).

Alongside these displayed behaviors was the unavoidable fact that a child might contract a sickness that would also prevent consumption: “Baby ill with chest infection and then stomach bug, consequently he had a very low appetite” (month 5, nonadherent caregiver).

Caregivers also reported that teething was a barrier to food consumption, especially during the middle period.

**Fig 4:**
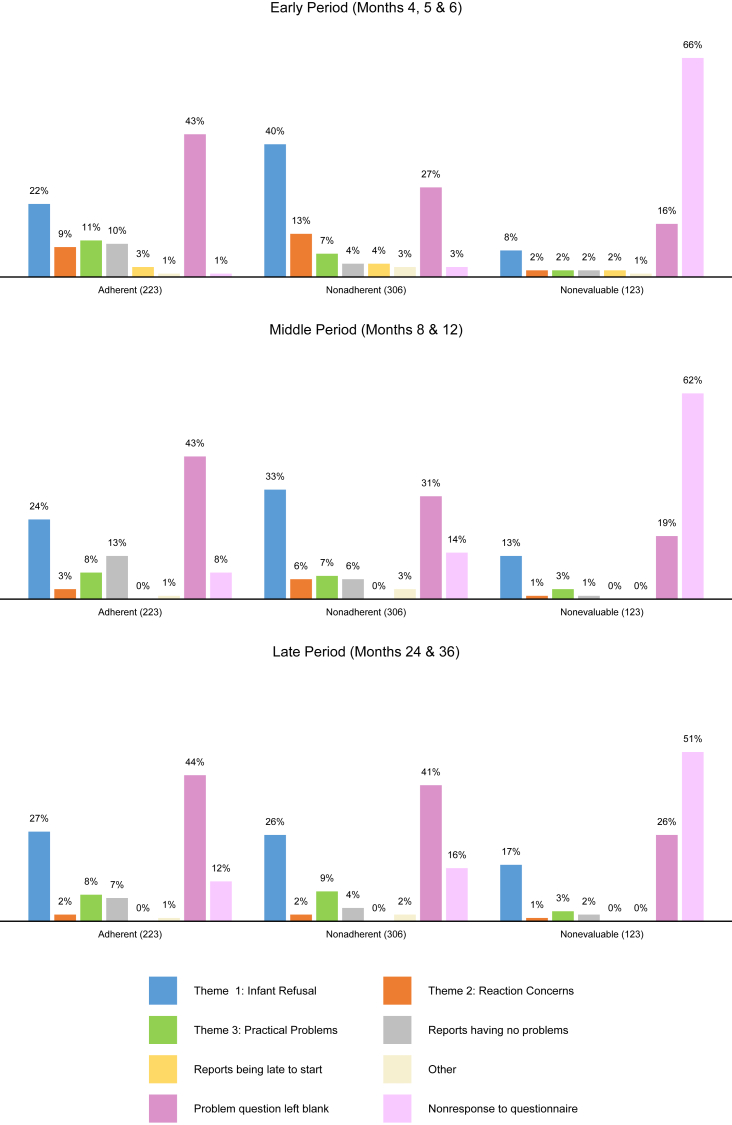
Distribution of thematic responses across time periods by EIG adherence status. Among adherent, nonadherent, and nonevaluable EIG participants, respectively, all qualitative codes recorded by these families in each time period (early period, middle period, and late period) were counted and totaled. *Bars* depict the relative distribution of thematic responses for each EIG adherence group by each time period.

Reports of infant refusal were proportionally greater among nonadherent caregivers (40% of responses) than among adherent caregivers (22% of responses; χ^2^ = 52.8, *P* < .001) in the early period (Fig 4, top row). This difference diminished over time as adherent caregivers reported more problems with infant refusal and nonadherent caregivers reported less, with proportions becoming comparable in the late period (Fig 4, middle and bottom rows).

#### Theme 2: Concerns about perceived reactions and intolerances

Concern about perceived reactions to the allergenic foods was another significant challenge. The main categories within this theme were the caregivers showing concerns about (1) digestive issues, (2) skin issues, and (3) an actual allergy/intolerance. Caregivers often questioned the normality of these issues and needed further advice and support.

At the beginning of the study (Fig 3; early period, theme 2), caregivers were cautious about following recommendations if they thought the allergenic food was causing an adverse reaction in their child: “My baby seems to get very constipated when he eats egg, so I have not been giving him the guideline amounts.” (month 5, adherent caregiver).

Because of this uncertainty, caregivers wanted to hear from a specialist to resolve the issue: “My baby has developed eczema and I'm not sure if a rattling noise when breathing is wheezing or to do with a cold that she has, I did try to contact someone but I missed their call and have not had time to try to contact again…” (month 6, adherent caregiver).

As time passed, the overall prevalence of these concerns decreased (Fig 3; middle period and late period, theme 2) as caregivers received support and developed more certainty regarding attribution: “As advised, I have not given XXX egg as we believe it was the cause of his vomiting. Since he has not been given egg as a food in-itself he has not suffered from vomiting…” (month 8, nonadherent caregiver).

Concerns about reactions were proportionally greater among nonadherent caregivers (13% of responses) than among adherent caregivers (9% of responses; χ^2^ = 6.43, *P* = .0122) during the early period (Fig 4, top row). As with theme 1, this difference also diminished over time, with both adherent and nonadherent caregivers reporting less concerns about reactions (Fig 4, middle and bottom rows).

#### Theme 3: Practical problems

The final theme was EIG families experiencing practical problems associated with the regimen, which created an environment unconducive to success. The categories that represented this theme were as follows: (1) lifestyle inconveniences and (2) food preparation issues. These issues caused caregivers to question whether the benefits of the regimen outweighed the consequences associated with it.

As participants began the regimen, bringing the allergenic food to a palatable consistency and desired taste was an issue (Fig 3; early period, theme 3). Recipe ideas and advice about blending foods were provided on the study's Web site: “I have offered less tahini as I struggle to find food/recipes to serve with it. I have spoken to the dietician and I am going to download further recipe ideas from your website” (month 6, nonevaluable caregiver).

However, although families blended the allergenic foods, the total volume that the infant needed to consume often increased beyond a reasonable amount: “egg—very difficult to get into smooth consistency (the egg white), therefore the baby struggles to eat it and I cannot make the baby eat the whole recommended amount” (month 4, nonadherent caregiver).

Because of the increased volume of food, questions about overfeeding and breast milk reduction became a concern: “My only worry is that I might be feeding my baby too much food and he is not having much milk as a result and I wonder if he is getting enough nutrition” (month 6, adherent caregiver).

As participants continued with the regimen, food preparation issues subsided, and a wide range of lifestyle inconveniences arose (Fig 3; middle period and late period, theme 3), including family holidays and travel, caregivers returning to work, nurseries not allowing allergenic foods, supermarkets not carrying items, and required time management. As children developed more mature eating habits and began to eat alongside their families, continuing to introduce the allergenic food often became inconsistent with the family lifestyle: “I just can't get this food group in to our diet as a regular thing. None of us really eat much sesame and it's bottom of everyone's list.” (month 24, nonadherent caregiver).

Additionally, if another family member had a food allergy, incorporating allergenic foods into everyday familial food preparation became difficult, escalating the regimen's inconvenience (Fig 3; late period, theme 3).

Reports of practical constraints were actually proportionally less among nonadherent caregivers (7% of responses) compared with adherent caregivers (11% of responses; χ^2^ = 6.88, *P* = .009) during the early period (Fig 4, top row). Although an opposite trend to themes 1 and 2, these differences also appear to diminish by the end of the study (Fig 4, middle and bottom rows).

### Infant ethnicity, maternal age, and maternal quality of life at enrollment

We have previously shown that increased maternal age, infant nonwhite ethnicity, and lower maternal quality-of-life scores in the psychological domain at enrollment were significantly associated with EIG participants being nonadherent in the EAT study.^12^ In this article, during the early period (see Table E3 in this article's Online Repository at www.jacionline.org), older mothers were significantly more likely to report the dominant theme of infant refusal (RR, 1.16; 95% CI, 1.01-1.34; *P* = .03). There was a nonsignificant increase in reporting of infant refusal for both families of nonwhite infants (RR, 1.17; 95% CI, 0.98-1.39; *P* = .07) and mothers with lower quality of life at enrollment (RR, 1.11; 95% CI, 0.97-1.27; *P* = .12).

Additionally, older mothers were significantly more likely to describe being late to start the introduction (RR, 1.99; 95% CI, 1.14-3.49; *P* = .02), which was a significant determinant of per-protocol adherence in the EAT study.^2^ In contrast, mothers with lower quality of life at enrollment were less likely to report being late to start, although this difference was not significant (RR, 0.77; 95% CI, 0.47-1.27; *P* = .31).

## Discussion

In our quantitative analysis of factors associated with nonadherence with the EAT early introduction regimen, we identified specific enrollment and early postenrollment factors as being significant.^12^ In this article we have looked at the problems reported by EIG mothers during allergenic food introduction and ongoing consumption, irrespective of adherence status.

Three key themes were identified. First, the infant refused the allergenic food, causing a sense of defeat among caregivers. Second, caregivers were concerned that the allergenic foods might be causing a reaction in their child, causing need for advice and reassurance before consumption could recommence. Finally, the practical implications of the regimen compromised the ability of some caregivers to maintain consistent introduction. We have shown that these themes do correlate with adherence status and the factors identified with nonadherence in the quantitative analysis, including nonwhite ethnicity, maternal age, and maternal quality of life at enrollment.

With regard to theme 1 (ie, infant refusal), it has been shown that mature feeding and swallowing is a process of skill acquisition; only through trial and error during daily practice can oral motor movements become adequate after time.^13^ Issues with opening and closing the lips, movements of the tongue to transport food, initiation of swallowing, and sensory reactions, such as gagging or choking, do not signify overt immaturity but rather diminish over time through the sensory capability to tolerate new tastes and types of food. It was also shown that there was no significant correlation (*P* = .11) between age at the start of spoon feeding and weeks needed to develop the skill.^13^

Regarding taste, this was the predominant issue across all 3 periods. It is well established that all infants show characteristic taste preferences: sweet and savory elicit positive responses, and bitter and sour elicit negative responses.^14^ Early likes and dislikes are influenced by these innate preferences but are also modifiable. Repeated exposure to novel or disliked foods that occurs in a positive and supportive environment might promote the acceptance of and eventually a preference for those foods.^14^

It is plausible that EIG families perceived early rejection as overt infant refusal and were then tentative to persevere with offering the food. Although standard introduction group families were not given this open-text question, similar challenges with infant refusal during the commencement of complementary feeding can be assumed. A large qualitative evidence synthesis on parental experiences and perceptions of infant complementary feeding has been published.^15^ This found that, irrespective of age, parental worries, concerns, and confusion about the infant's developmental readiness for food and about the best way to feed infants persisted.^15^ These perceptions relate to a wider debate about whether there is a right age to introduce solids and, if so, when this is. This is an issue that has been long debated within the United Kingdom (UK).

Infant feeding recommendations in the UK have changed in recent years to state that allergenic foods need not be differentiated from other solid foods and that their deliberate exclusion beyond 6 to 12 months of age might increase the risk of allergy.^16^ This contrasts with the previous advice that allergenic food introduction after 6 months of age is entirely at parental discretion. This change in recommendations, although reflective of new evidence, might lead to confusion among parents.^15^ However, evidence that new guidelines can result in changes in infant feeding behavior has been shown in Australia in the HealthNuts cohort, in which publication of the revised Australian allergy guidelines removing the delay in introduction of allergenic foods occurred midway through recruitment.^17^ Families recruited after the new guidelines were more likely to introduce solids, including egg, earlier.

In addition to recommendations and guidelines, families also depend on health care professionals for infant feeding advice. This also has been of variable consistency, with a recent survey of pediatricians, immunologists, and family physicians in Canada finding that new guidelines regarding early introduction of allergenic foods were not constantly applied in clinical practice. Family physicians commonly recommended introduction of allergenic solids at age 1 year or more, whereas specialist pediatricians and allergists were more aligned with current recommendations.^18^ Without clear communication about updated policy guidelines and consistently applied clinical support, it will be challenging to encourage caregivers to persevere through the infant refusal associated with an early introduction regimen.

With regard to theme 2, (ie, concerns about perceived reactions), allergenic foods have historically been seen as dangerous foods to give infants. In the UK Infant Feeding Survey of 2010, mothers of 8- to 10-month-old babies were asked whether there were any particular ingredients they avoided giving their babies. Nearly half (45%) mentioned at least 1 ingredient.^1^ Among the allergenic foods recorded in those mentioning an ingredient, nuts were dominant (41%), with much smaller numbers avoiding eggs (12%) and dairy products (11%). It might be that if an allergenic food becomes more widely consumed by infants,^19^ as with peanuts in Israel,^20^ confidence in its safety will increase, with an associated reduction in perceived reactions to food consumption. Certainly, in the EAT study it was shown that the proportion of EIG families reporting potential reactions to foods in the key early introduction period far exceeded the percentage of EIG infants who actually had an allergy to these foods (eg, 33 EIG families reported IgE-type symptoms to milk, but only 2 EIG infants had a milk allergy).^2^ Early allergenic food introduction in the EAT study was found to be safe, and widespread consumption of peanut by Israeli infants has not been associated with a single fatality in Israel between 2004 and 2011.^21^ Nonetheless, families in the EAT study often sought advice to have the confidence to continue with introduction. Although preventing food allergies through early allergenic food introduction would decrease the significant familial and health system costs associated with childhood food allergies,^22^ the regimen, if perceived as proscriptive, might also medicalize food introduction, presenting new costs, such as additional support needed from dieticians and widespread training of health professionals.

With regard to theme 3, it was clear that bringing foods to a desired consistency, presenting foods in a palatable way, and adopting a consistent routine were persistent challenges. To overcome these inconveniences, companies have already been formed with the aim of producing infant meals specifically containing food allergens. These range from products containing just peanut, in some cases using the dosing demonstrated to induce tolerance in the LEAP study and EAT per-protocol population. Other products include multiple allergens (eg, one product includes peanut, milk, shellfish [shrimp], tree nuts [almond, cashew, hazelnut, pecan, pistachio, and walnut], egg, fish [cod and salmon], grains [oat and wheat], soy, and sesame), although the individual dose of allergen protein in such products is unlikely to reach the threshold to induce tolerance as demonstrated in the EAT study. Historically, food companies would not have considered incorporating allergenic foods such as peanut into an infant meal. Potentially some of these products, assuming the dose is sufficient to induce tolerance, may have a role to play in overcoming difficulties with palatability, preparation and sourcing allergens. However, their potential impact on broader dietary requirements and breastfeeding are unknown. Furthermore, they are likely to impose an additional financial cost on families if consumption is continued.

As seen in Fig 3, the inconvenience of maintaining the allergenic feeding regimen was more dominant in the late period. This was often due to the allergenic food not being a food routinely consumed in the home. Over time, families might lose motivation as the additional burden of continuing consumption of the allergenic food could start to outweigh the perceived benefits. However, one significant consideration parents make when deciding what to feed children after complementary feeding has commenced is evaluating the perceived healthiness of the specific food.^15^ If the allergenic food can be marketed not only for its potential to prevent food allergies but also for its healthiness, decision making might be influenced. Understanding how messaging and incentives in the infant feeding environment can influence behavioral choice might be useful to consider for future implementation strategies.

A potential weakness of this study is the extent to which responses to a question about problems introducing allergenic foods in a highly self-selected group of families participating in a randomized controlled trial might relate to the issues likely to be encountered with early food introduction in the real world. Although we acknowledge that additional challenges can occur, this analysis has identified issues that will likely pertain regardless of context, such as initial infant refusal, taste preferences, and infant ailments, and will require addressing.

An additional concern is that the wording of the problem question, “a particular problem with your baby consuming the foods,” might have biased responses to be about challenges at the point of consumption. Using interviews or focus groups as a method of qualitative data collection might have allowed challenges to be revealed beyond consumption. Finally, it should be noted that although caregivers were free to type in as much as they wanted into the text box, the storage system cut off responses at around 200 characters. Although a limitation, those who wrote more than the allotted text were found to have mentioned their key concern early within the response, and a principal theme could still be derived.

We did not undertake a food-specific analysis in which we attempted to relate the identified themes to individual foods. There were 2205 free text responses analyzed for this article, and many responses mentioned multiple foods in both positive and negative contexts. Although such an analysis might have identified certain themes as predominating with specific foods, if the early introduction of multiple allergenic foods is to be recommended, the advice given is very unlikely to be food specific. Instead, it would address holistically the themes we have identified in this article and how these can be overcome.

Introducing allergenic foods early in infancy is effective in preventing the development of food allergy and has resulted in changes to infant feeding guidelines.^23^ Addressing the challenges identified in this article will facilitate the sustainable adoption and implementation of this evidence-based intervention.

**Clinical implications**

Understanding problems experienced with allergenic food introduction and sustained consumption can help overcome adherence barriers. Specific communication and support strategies to address these problems might be advantageous in practice.

## Supplementary data

Online Repository text

Fig E1

Fig E2
