# Supplementary material for: Challenges experienced with early introduction and sustained consumption of allergenic foods in the Enquiring About Tolerance (EAT) study: A qualitative analysis
Source: J Allergy Clin Immunol. 2019 Dec;144(6):1615–23. doi: 10.1016/j.jaci.2019.09.004 (PMC6904907; doi:10.1016/j.jaci.2019.09.004)
Supplement: Online Repository text [file mmc1.docx]

**Supplementary Appendix**

**Challenges experienced with the early introduction and sustained consumption of allergenic foods in the EAT study: a qualitative analysis**

**Paula Voorheis, MSc,^a^ Sadie Bell, PhD,^b^ Laura Cornelsen, PhD,^b^ Matthew Quaife, PhD,^b^ Kirsty Logan, PhD,**^c^ **Tom Marrs, MB BS,^c^ Suzana Radulovic, MD,^c^ Joanna Craven, MPH,^c^ Carsten Flohr, PhD,^d^ Gideon Lack, M.B. B.Ch,**^c^ **and Michael R. Perkin, PhD,**^e^ **on behalf of the EAT Study Team**

From the ^a^the Executive Office, London School of Hygiene and Tropical Medicine, ^b^the Faculty of Public Health and Policy, London School of Hygiene and Tropical Medicine, ^c^the Paediatric Allergy Research Group Department of Women and Children’s Health, School of Life Course Sciences, King’s College London, ^d^the Unit for Population-Based Dermatology Research, St John’s Institute of Dermatology, School of Basic and Medical Biosciences, Faculty of Life Sciences & Medicine, King’s College London, ^e^the Population Health Research Institute, St George's, University of London

**Methods**

*Dietician advice booklets*

At enrolment the EIG families received a “Baby’s First...!” booklet which included basic introduction tips & recipes for a three to five month old baby. In the 5, 6 and 7 month interim questionnaires, EIG families were encouraged to download an “Early Introduction Group Follow On Tips & Recipes” booklet from the EAT Study website with the following message:

**Please remember that the ‘FOLLOW ON Tips and Recipes’ booklet is available to download from the links page of the EAT website:**

**http://www.eatstudy.co.uk/links**

**This aims to give you guidance on the weaning process, as well as some different textures and flavours you can offer your growing baby over the coming months.**

**You will also find quick and easy EAT recipes that combine at least 2 or 3 key foods at a time, which makes it easier to prepare and offer them on a weekly basis!**

When the families attended the one year clinic visit, EIG families were given a "Key Food Options & Portions" booklet. This suggested a variety of forms in which the required EAT allergen amount could be given to a one year or older infant. Also at the one year visit, both groups were given a booklet by the dieticians giving generic advice about healthy eating for toddlers: “Eating Well for 1—5 Year Olds”.

*Dietician produced videos*

An EIG parent (usually the mother) watched a video at the enrolment visit to help explain how best to introduce the allergenic foods. This was available on line to be watched again (as noted in the “Baby’s First...!” booklet which contained the recommendation to "Go to the ‘Video’ section of the EAT Study Website to watch the Early Introduction Group Video Clips!" Online the video was too long to stream as one video and was divided up into a number of sections for families to watch: Introduction; Cow’s Milk Yoghurt; Egg; Fish; Peanut; Sesame; Wheat; Closing Points; Supporting Resources For Baby; and Supporting Resources For Mum.

*Automatic alert emails to EAT study dieticians*

If a consumption alert (see below) had been generated and displayed within the Interim Questionnaire, this also led to an automated email being sent to the EAT study dieticians informing them of the exact amount of all six early introduction foods that the family had reported their infant consuming in that particular interim questionnaire. They could then contact the family to see if there were any problems and offer some help. Such communications with families were logged in a database maintained by the EAT Study dieticians to provide continuity of support. The automated email sent to the dieticians also included the response a family had given (if they had given one) to the problem question: "If you have had a particular problem with your baby consuming the foods over the past month please provide brief details in the following box."

A separate alert was generated if a mother had not started giving her infant a specific allergenic food at all when she completed the questionnaire, again including the response to the “any particular problem” question. There were also email alerts to the dieticians if a mother had stopped breastfeeding and one for if the infant had started being fed formula milk.

*EAT Study team contact details*

Participating families were given a dedicated EAT email address and a dedicated EAT study telephone number to contact at any time during their child's participation on the study.

*EIG consumption monitoring and alert in the Interim Questionnaires*

Each interim questionnaire contained a table to remind mothers what the weekly guideline amounts were for the key foods and giving some examples of the form of food that this would be the equivalent of:

**The following table is to help remind you what the weekly guideline amounts (100%) are for some examples of the key foods and what 25%, 50% and 75% of these amounts would look like.**

**
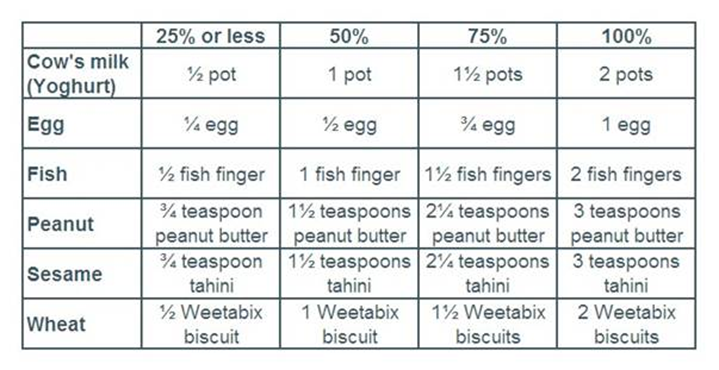
**

Following this table were the degree of adherence questions for each of the six foods: was the infant having 25% or less, 50%, 75% or 100% of the weekly target, or not tried yet, and families had to give an answer for each of the six foods for each week in the interim questionnaires through to one year of age or for each month in the interim questionnaires beyond one year of age. Only if the consumption for one or more foods was 50% or less of the recommended amount did this message come up:

**Alert!**

**Your baby has eaten 50% or less of:**

**Cow's milk on {V2} weeks**

**Egg on {V3} weeks**

**Fish on {V4} weeks**

**Peanut on {V5} weeks**

**Sesame on {V6} weeks**

**Your baby may be avoiding a food because of an allergy or a feeding problem that we are aware of.**

**In all other circumstances we think it is important that in order to protect your baby from developing a food allergy that it is during these early months that your baby should be eating the full weekly guideline amount of the intervention foods by following the plan on the Star Chart you were given.**

**Follow your baby's appetite and remember that you can split the weekly guideline amounts into smaller servings given over the week if necessary.**

**Please contact us if you are having difficulties feeding the foods to your baby or you suspect your baby is having a problem with a food that you have not told us about.**

**Otherwise, we encourage you to try to ensure your baby is consuming the weekly guideline amounts.**

**TABLE E1. Overall per-protocol adherence criteria in the EAT study**

| **Adherence definitions** | **Adherence evaluable children meeting the adherence definitions** |
| --- | --- |
| **Standard Introduction Group (SIG)**  *(N=606/651 children adherence evaluable)** |  |
| - Criterion A: Exclusive breastfeeding for at least three months duration (water and/or oral rehydration solution allowed) | 100% (606/606) (A)  12.0% have had water by 3 months of age |
| - Criterion B: Continued breastfeeding up to five months of age | 99.7% (604/606) (B) |
| - Criterion C: No consumption of peanut, egg, sesame, fish or wheat before five months | 97.4% (590/606) (C) |
| - Criterion D: No introduction of cow’s milk formula (or goat’s milk formula) (or consumption of less than 300 mls/day) between three months and six months of age | (1) No formula pre six months 85.6% (519/606)  (2) Consumption of less than 300mls/day 8.8% (53/606)†  (1) or (2) 94.4% (572/606) (D)  †median age of introduction of 22 weeks |
| **Overall SIG per-protocol adherence (meets all criteria)** | **92.1% (558/606) (A, B, C & D)** |
| **Early Introduction Group (EIG)**  *(N=529/652 children adherence evaluable)** |  |
| - Criterion A: Exclusive breastfeeding for three months duration (water and/or oral rehydration solution allowed) | 100% (529/529) (A)  13.1% have had water by 3 months of age |
| - Criterion B: Continued breastfeeding up to five months of age | 99.6% (527/529) (B) |
| - Criterion C: Consumption of at least five of the allergenic foods in at least 75% of the recommended amount (3g allergen protein/week), for at least five weeks between three months and six months of age | 42.3% (224/529) (C) |
| **Overall EIG per-protocol adherence (meets all criteria)** | **42.2% (223/529) (A, B & C)** |

* Adherence status non-evaluable for 7% (45/651) of the SIG and 19% (123/652) of the EIG participants

**TABLE E2. Enrolment demographic data**

|  |  | **SIG**  % (n/N) | **EIG**  % (n/N) |
| --- | --- | --- | --- |
| **Number in group** |  | 651 | 652 |
| **Demography** |  |  |  |
| Mean age at enrollment (months)  (SD) |  | 3.39 (n=651)  (0.24) | 3.38 (n=652)  (0.22) |
| Mean age at three year visit (months)  (SD) |  | 37.9 (n=601)  (3.3) | 37.9 (n=572)  (3.4) |
| Sex: | Male  Female | 52.1 (339/651)  47.9 (312/651) | 48.2 (314/652)  51.8 (338/652) |
| Ethnicity: | White  Black  Asian†  Chinese  Mixed | 84.0 (547/651)  2.9 (19/651)  1.7 (11/651)  0.5 (3/651)  10.9 (71/651) | 85.4 (557/652)  3.4 (22/652)  2.6 (17/652)  1.2 (8/652)  7.4 (48/652) |
| Pet ownership |  | 44.6 (290/650) | 40.6 (264/651) |
| Maternal education:  (age at leaving full-time education) | ≤16  17-18  >18 | 6.2 (40/650)  13.7 (89/650)  80.2 (521/650) | 5.2 (34/652)  12.7 (83/652)  82.1 (535/652) |
| **Smoking** |  |  |  |
| Maternal |  | 3.1 (20/650) | 3.4 (22/651) |
| Paternal |  | 10.9 (71/650) | 10.8 (70/651) |
| **Family history** |  |  |  |
| Median maternal age (years): |  | 33 (n=650)  (range 19 – 46) | 33.5 (n=652)  (range 19 – 45) |
| Siblings | 0  1  2  3+ | 38.3 (249/651)  36.9 (240/651)  16.4 (107/651)  8.5 (55/651) | 37.3 (243/652)  39.3 (256/652)  14.9 (97/652)  8.6 (56/652) |
| **Birth history** |  |  |  |
| Mean birth weight grams  (SD) |  | 3560 (n=651)  (487) | 3570 (n=651)  (489) |
| Mode of delivery:* | Vaginal  Caesarean | 77.3 (503/651)  22.7 (148/651) | 72.4 (472/652)  27.6 (180/652) |
| Mean gestational age (weeks) |  | 39.7 (n=651) | 39.9 (n=652) |
| **Participant enrollment atopy status** |  |  |  |
| Skin-prick test positive (>0 mm) |  | N/A | 5.1 (33/652) |
| Visible eczema |  | 24.2 (157/650) | 24.5 (160/652) |
| Median SCORAD  (infants with eczema) |  | 7.5 (n=157)  (range 3.5 – 49.2) | 7.5 (n=160)  (range 3.5 – 75.0) |
| **EIG median age of allergenic food first consumption**  **(weeks)** |  |  |  |
| Dairy  Egg  Fish  Sesame  Peanut  Wheat |  | -  -  -  -  -  - | 17.3  19.6  19.6  19.6  19.6  20.6 |
| **Family atopy status (self-reported)** |  |  |  |
| ***Maternal*** |  |  |  |
| Eczema |  | 34.2 (222/650) | 34.9 (227/651) |
| Asthma |  | 26.8 (174/650) | 25.8 (168/651) |
| Maternal atopy‡ |  | 63.2 (411/650) | 61.9 (403/651) |
| ***Paternal*** |  |  |  |
| Eczema |  | 21.1 (137/650) | 18.9 (123/651) |
| Asthma |  | 23.5 (153/650) | 21.8 (142/651) |
| Paternal atopy‡ |  | 55.7 (362/650) | 50.5 (329/651) |
| **Maternal allergenic food consumption** |  |  |  |
| During pregnancy |  | 100.0 (639/639) | 100.0 (631/631) |
| During breastfeeding |  | 100.0 (639/639) | 100.0 (631/631) |

* P < 0.05

† Asian refers to Indian, Pakistani and Bangladeshi

‡Eczema, asthma or hay fever

**TABLE E3. Risk of reporting qualitative themes by factors previously shown to be linked to non-adherence during the Early-Period**

| **Independent Variables** | **Dependent Variables** | | | |
| --- | --- | --- | --- | --- |
|  | **Theme 1**  Infant Refusal | **Theme 2**  Concerns about Reactions | **Theme 3**  Practical Problems | Reports being Late to Start |
|  | **RR (95% CI)** | **RR (95% CI)** | **RR (95% CI)** | **RR (95% CI)** |
| **Infant Ethnicity** | | | | |
| Non-White Ethnicity | 1.17 (0.99-1.39) | 1.28 (0.87-1.89) | 0.76 (0.44-1.33) | 1.04 (0.50-2.18) |
| **Maternal Age** | | | | |
| ≥33 years (median) | 1.16 (1.01-1.34)* | 1.02 (0.76-1.36) | 0.94 (0.69-1.29) | 1.99 (1.14-3.49)* |
| **Maternal Quality of Life†** | | | | |
| <16 (median) | 1.11 (0.97-1.27) | 1.09 (0.82-1.45) | 1.03 (0.75-1.42) | 0.77 (0.47-1.27) |

*P<0.05 † Psychological domain

Risks are calculated in the Early-Period and are only estimated among participants who responded to all three questionnaires in this time period.

**Supplementary Appendix Figure Legends**

**Fig E1. Interim questionnaire completion by study group**

**Fig E2. Sankey plot of the fow in type of respnses to the problem question over time**

At each interimquestionniare time point the distribution of three possible response types is given. The Sankey plot shows the relationship with the response in the subsequent interim questtionnaire. Consdierable flux is seen in response type. Superimposed is the colour coded adherence status. Participants who left the problem question blank in the key 4, 5 & 6 month interim questionnaires were much more likely to be adherent (red) as opposed to those who entered a response (blue/purple). Those who did not complete these key interim questionnaires (no data) were most likely to have been non-adherent (blue). Over time, whether familes were entering a response or leaving the problem question blank was much less closely related to adherence status (both the same shade of purple).

The 7 month interim questionnaire is excluded for the reasons given in the footnote to Figure 1 and the 12 month and 36 month data are excluded because these visits coincided with clinic visits distorting the pattern observed in the Figure from the on line questionnaire data only.
