# Supplementary figures and images for: Challenges experienced with early introduction and sustained consumption of allergenic foods in the Enquiring About Tolerance (EAT) study: A qualitative analysis
Source: J Allergy Clin Immunol. 2019 Dec;144(6):1615–23. doi: 10.1016/j.jaci.2019.09.004 (PMC6904907; doi:10.1016/j.jaci.2019.09.004)

Percentage

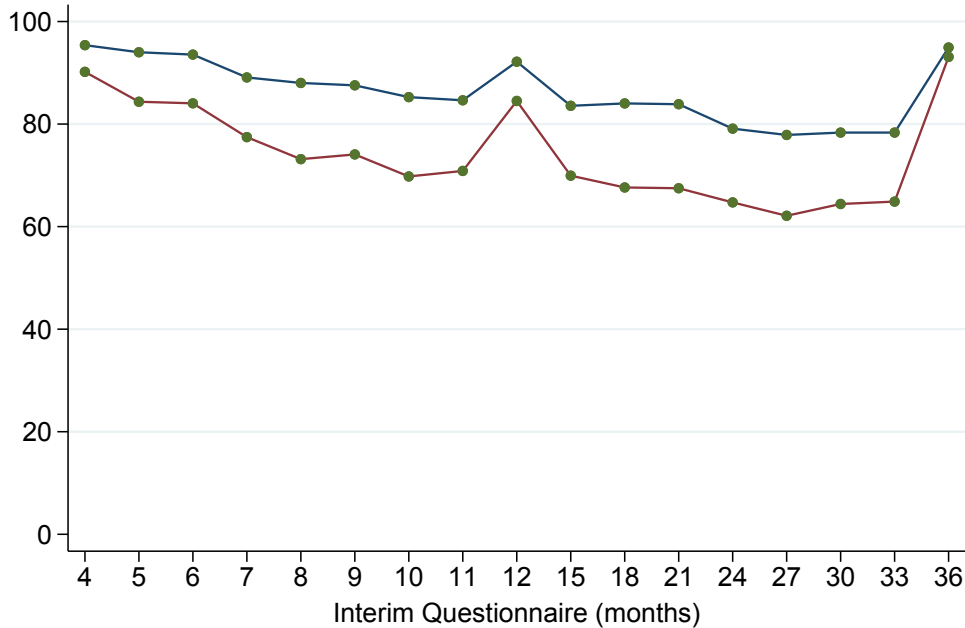

Standard introduction

Early introduction

Supplement: Fig E1 [file mmc2.pdf]

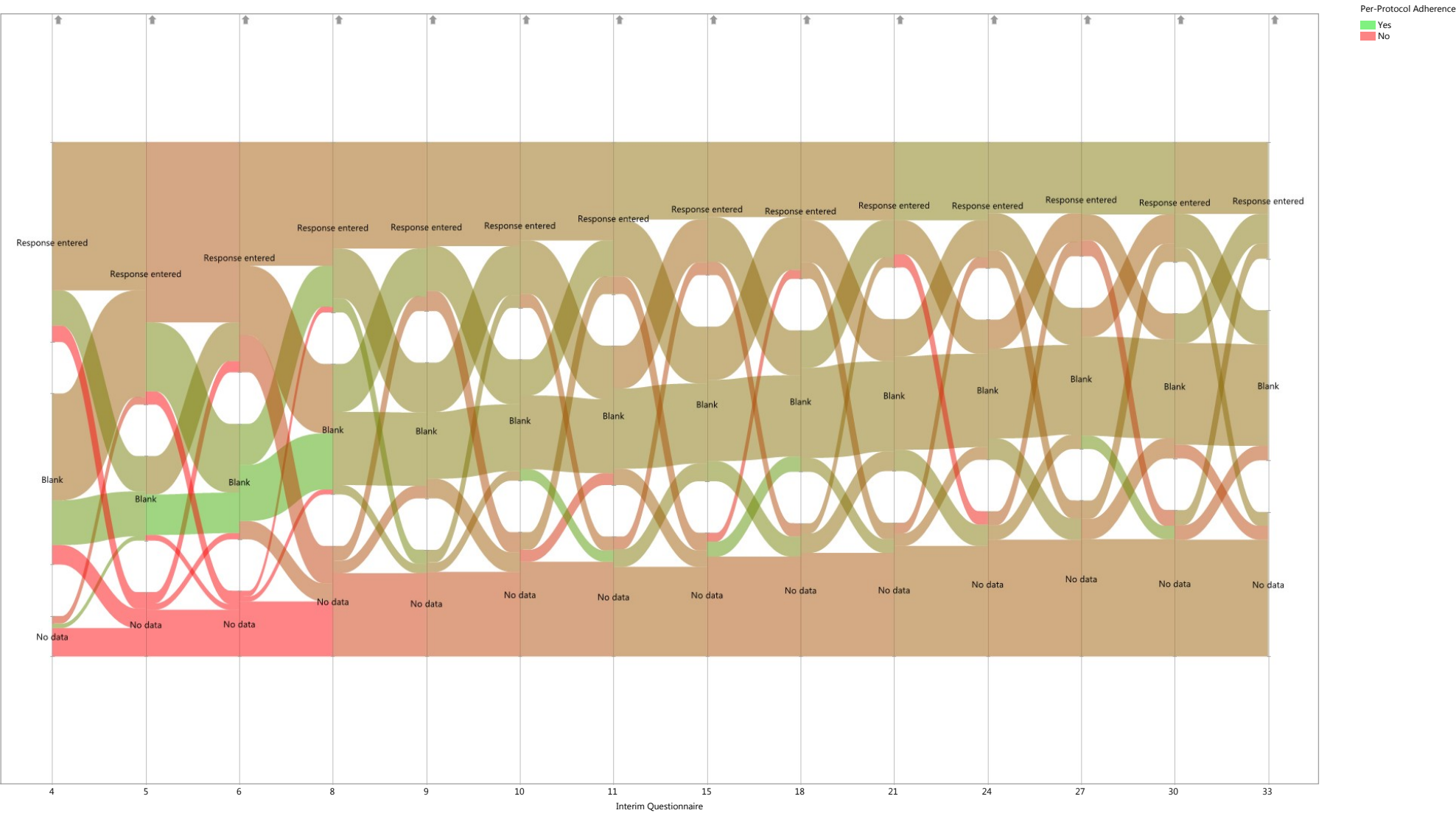

Supplement: Fig E2 [file mmc3.pdf]
